# Illustration of different modelling assumptions for estimation of loss in expectation of life due to cancer

**DOI:** 10.1186/s12874-019-0785-x

**Published:** 2019-07-09

**Authors:** Therese M.-L. Andersson, Mark J. Rutherford, Paul C. Lambert

**Affiliations:** 1 Department of Medical Epidemiology and Biostatistics, Karolinska Institutet, Box 281, Stockholm, 17177 Sweden; 2 Department of Health Sciences, University of Leicester, Leicester, UK

**Keywords:** Life expectancy, Loss in life expectancy, Cancer, Survival, Relative survival

## Abstract

**Background:**

The life expectancy of cancer patients, and the loss in expectation of life as compared to the life expectancy without cancer, is a useful measure of cancer patient survival and complement the more commonly reported 5-year survival. The estimation of life expectancy and loss in expectation of life generally requires extrapolation of the survival function, since the follow-up is not long enough for the survival function to reach 0. We have previously shown that the survival of the cancer patients can be extrapolated by breaking down the all-cause survival into two component parts, the expected survival and the relative survival, and make assumptions for extrapolation of these functions independently. When extrapolating survival from a model including covariates such as calendar year, age at diagnosis and deprivation status, care has to be taken regarding the assumptions underlying the extrapolation. There are often different alternative ways for modelling covariate effects or for assumptions regarding the extrapolation.

**Methods:**

In this paper we describe and discuss different alternative approaches for extrapolation of survival when estimating life expectancy and loss in expectation of life for cancer patients. Flexible parametric models within a relative survival setting are used, and examples are presented using data on colon cancer in England.

**Results:**

Generally, the different modelling assumptions and approaches give small differences in the estimates of loss in expectation of life, however, the results can differ for younger ages and for conditional estimates.

**Conclusion:**

Sensitivity analyses should be performed to evaluate the effect of the assumptions made when modelling and extrapolating survival to estimate the loss in expectation of life.

## Introduction and Background

A useful summary measure for survival data is the mean survival time, or life expectancy, as an alternative to survival proportions at selected time points. The life expectancy from the date of cancer diagnosis until death (irrespective of cause of death), gives an estimate of average the number of years cancer patients are expected to live after they are diagnosed with cancer [1, 2]. By contrasting the life expectancy among cancer patients to the life expectancy amongst similar people in the general population, one can estimate the loss in expectation of life due to cancer [3]. This can be expressed as a proportion of expected life lost by dividing the difference by the expectation of life. The loss in expectation of life, or the proportion of expected life lost, are useful measures for quantifying the cancer burden in the society and differences in survival between groups. But it can also be quantified at an individual level and interpreted as the average number of life years a cancer patient is expected to lose due to the cancer diagnosis, and can therefore be an important measure in clinical research and useful for understanding the impact of a cancer diagnosis on an individual’s life expectancy [4–6]. This measure, although theoretically easy to estimate, generally requires extrapolation of both the expected (general-population) survival and the observed all-cause survival (of the cancer patients), due to limited follow-up. The expected survival can be extrapolated by making assumptions about the future mortality in the general population. We have previously shown that the survival of the cancer patients can be extrapolated by breaking down the all-cause survival into two component parts, the expected survival and the relative survival, and make assumptions for extrapolation of these functions independently [7]. For most types of cancer the excess mortality is low after some years from diagnosis, so the expected mortality dominates for long-term follow-up, and the extrapolation therefore mostly depends on the extrapolation of the expected survival. This approach has previously been proposed by Hakama and Hakulinen, by using grouped data (a life tables of relative survival), by assuming that the cancer patients have a constant excess hazard after the available follow-up [3]. Others have proposed methods where the cancer patients are assumed to have the same mortality as the general population (excess mortality is zero) after the observed follow-up [1, 8], or extrapolated survival based on a linear regression of some functiont of the survival [9]. We proposed a modelling approach, using flexible parametric models, where the assumption of a constant, or zero, excess hazard is not required [7].

When extrapolating survival from a model including covariates such as calendar year, age at diagnosis and deprivation status, care has to be taken regarding the assumptions underlying the extrapolation. There are often different alternative ways for modelling covariate effects or for assumptions regarding the extrapolation. In this paper we will describe and discuss a few of the different options, and show examples using data on colon cancer in England.

**Fig. 1:**
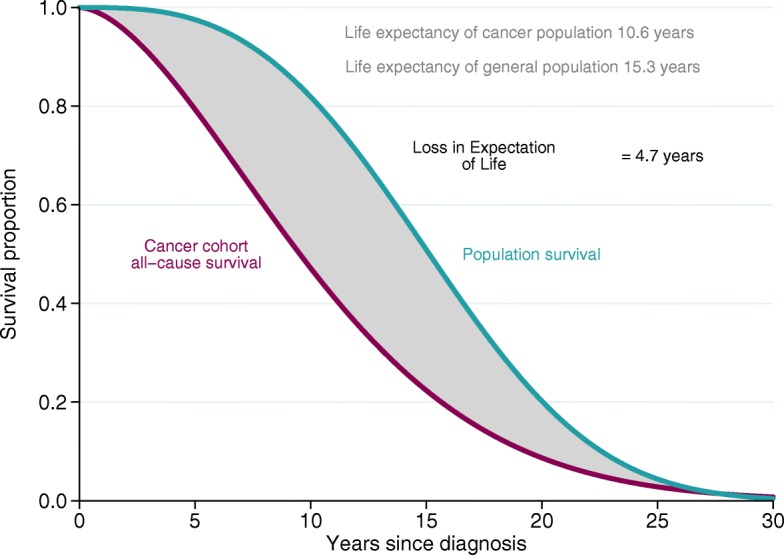
Illustration of loss in expectation of life, LEL

The loss in expectation of life (LEL) due to cancer is the difference between the expectation of life the patients would have had if they had not been diagnosed with cancer, estimated using population mortality rates for the general population, and the observed expectation of life among the cancer patients, as illustrated in Fig. 1 and Eq. 1. *S*^∗^(*t*) is the expected survival, usually obtained from population life tables. The overall, or all-cause, survival, *S*(*t*), can vary by values of the covariates, ***z***, which can, for example, be patient characteristics such as sex, age and calendar year of diagnosis and/or tumour characteristics such as stage or grade. The expected survival is allowed to vary by the factors $\phantom {\dot {i}\!}\boldsymbol {z^{'}}$, on which the population mortality rates are stratified, which usually represent a subset of ***z***, typically age, sex and calendar year.

1 $$ LEL(\boldsymbol{z})=\int\nolimits_{0}^{t_{max}}S^{*}(u;\boldsymbol{z^{'}})du-\int\nolimits_{0}^{t_{max}}S(u;\boldsymbol{z})du,   $$

where *t*=0 is the time of diagnosis. Integration should in theory be done up to *∞*, but in practice a time point, *t*_*max*_, is used where the survival function is assumed to have reached zero (i.e. all patients are assumed dead). It can also be of interest to estimate conditional LEL, conditioning on survival up to a certain point, *t*_*c*_,

2 $$ LEL(\boldsymbol{z}|T>t_{c})=\int\nolimits_{t_{c}}^{t_{max}}\frac{S^{*}(u;\boldsymbol{z^{'}})}{S^{*}(t_{c};\boldsymbol{z^{'}})}du-\int\nolimits_{t_{c}}^{t_{max}}\frac{S(u;\boldsymbol{z})}{S(t_{c};\boldsymbol{z})}du.   $$

Another measure of interest is the proportion of expected life lost (PELL), given by Eq. 3,

3 $$ PELL(\boldsymbol{z})=\frac{\int\nolimits_{0}^{t_{max}}S^{*}(u;\boldsymbol{z^{'}})du-\int\nolimits_{0}^{t_{max}}S(u;\boldsymbol{z})du}{\int\nolimits_{0}^{t_{max}}S^{*}(u;\boldsymbol{z^{'}})du}.   $$

To get a measure of the impact a cancer has on a population level the LEL for each individual, *j*, in a cohort of *N* individuals can be summed;

4 $$ \sum\limits_{j=1}^{N}LEL(\boldsymbol{z}_{j}).   $$

This gives the total amount of person-years lost in the population.

**Fig. 2:**
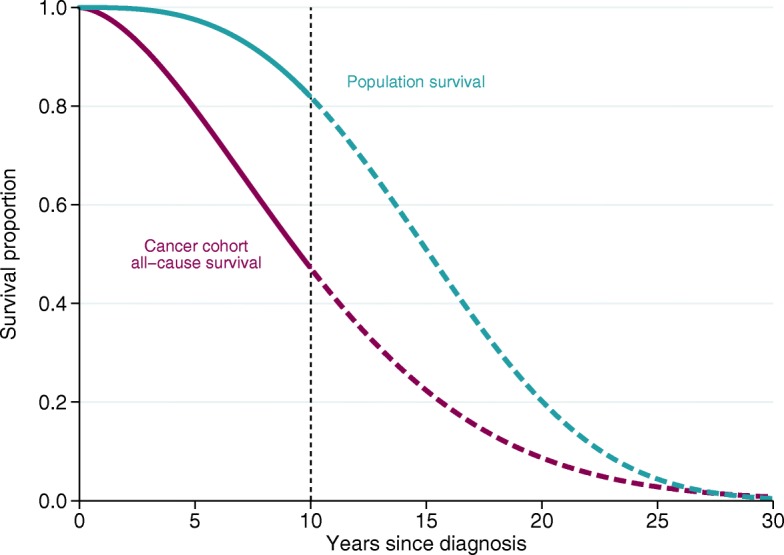
Illustration of extrapolation of survival functions

The difficulty in estimating these quantities is that we rarely have follow-up until the time point when all individuals have died, and therefore need to extrapolate the survival functions, as illustrated in Fig. 2. The expected survival function can be estimated by making assumptions about the future mortality rates in the population, which are usually easily available, but it is difficult to extrapolate the all-cause survival among the cancer patients. Extrapolations of the all-cause survival can be based on a parametric distribution, but it is difficult to find a parametric model that fits the observed data and also capture the mortality beyond the available data well enough for reliable extrapolation. By breaking down the all-cause survival into two component parts, the expected survival and the cancer survival, one can extrapolate these two component separately. Since the mortality due to cancer decreases with time for most cancers, and is low for long-term follow-up, the expected mortality dominates in the extrapolation. We have previously shown that extrapolations of all-cause survival among cancer patients is possible by extrapolating the cause-specific survival using a relative survival framework.

### Relative survival

Relative survival [10, 11] is the method of choice for estimating cancer patient survival, as it does not rely on correct classification of cause of death. It is defined as the observed (all-cause) survival among the cancer patients divided by the expected survival the patients would have experienced had they not had cancer.

In the relative survival setting, the all-cause survival, *S*(*t*), as a function of time *t* since diagnosis, can be written as

5 $$ S(t;\boldsymbol{z})=S^{*}(t;\boldsymbol{z^{'}})R(t;\boldsymbol{z}),   $$

where *R*(*t*) represents the relative survival. The hazard analogue of relative survival is excess hazard, and it measures the mortality the patients experience in excess of what would have been expected if they had not had cancer. The overall, all-cause, hazard, *h*(*t*), among the patients is written as the sum of the expected hazard, *h*^∗^(*t*), and the excess hazard, *λ*(*t*), associated with the cancer

6 $$ h(t;\boldsymbol{z})=h^{*}(t;\boldsymbol{z^{'}})+\lambda(t;\boldsymbol{z}).   $$

The relative survival can be estimated using a modelling approach that enables flexible modelling of the baseline excess hazard [11], and one such model is the flexible parametric survival model [12, 13]. The flexible parametric survival model [12, 13] uses restricted cubic splines to model the baseline cumulative hazard. The use of splines enables the model to capture complex baseline cumulative hazard functions, and gives a parametric model without the need of strong distributional assumptions. The flexible parametric survival model was first introduced by Royston and Parmar in 2001 [12, 14]. The model has also been extended for relative survival, by modelling the log cumulative excess hazard using restricted cubic splines [13, 15]. In a flexible parametric survival model for relative survival, the log cumulative excess hazard, ln*Λ*(*t*;***z***), is modelled as a function of follow-up time, *t*, using splines as:

7 $$ \ln (\Lambda(t;{\boldsymbol{z}}))=s(x;\boldsymbol{\gamma_{0}})+{\boldsymbol{z}}\boldsymbol{\beta},   $$

where *x*= ln(*t*),*s*(*x*;***γ***_***0***_) is a restricted cubic spline function and ***z*** are covariate effects. Time-varying effects (non-proportional hazards) can easily be modelled by adding interaction terms by a covariate and a new set of spline variables for time. The relative survival can easily be obtained using the relationship between the survival and the cumulative hazard function,

8 $$ R(t;{\boldsymbol{z}}) = \exp(-\Lambda(t;{\boldsymbol{z}})).   $$

### Approaches for extrapolation of relative survival

Based on a flexible parametric relative survival model the extrapolated relative survival can be estimated. There are different approaches that can be used for extrapolating the relative survival, by making different assumptions about the excess mortality beyond the point of available data. The two most common approaches are to assume population cure [16] or to extrapolate based on the model parameters. Population cure is when the mortality rate of the diseased patients return to that expected in the population, i.e. the excess hazard goes to zero. When the full (extrapolated) relative survival function has been estimated the full (extrapolated) all-cause survival function can be estimated by multiplying the relative survival by the (extrapolated) expected survival function [7]. The LEL is then estimated as

9 $$ LEL(\boldsymbol{z})=\int\nolimits_{0}^{t_{max}}S^{*}(u;\boldsymbol{z^{'}})du-\int\nolimits_{0}^{t_{max}}R(u;\boldsymbol{z})S^{*}(u;\boldsymbol{z^{'}})du.   $$

We have shown that estimating the LEL using Eq. 9 performs much better than using Eq. 1 [7].

## Methods

To illustrate different assumptions and approaches that can be used when estimating LEL, National Cancer Registry Data provided by Public Health England was used. We used data on colon cancer (International Classification of Diseases–10 site code C18) in England in the years 1998-2013. The dataset contains all individuals diagnosed with colon cancer in England during the specified years, and holds information about the individual as well as information about the cancer diagnosis. We used information on age at diagnosis, calendar year of diagnosis, time between diagnosis and death or censoring, as well as a categorical variable of deprivation status at diagnosis based on postcode areas split into five categories. Follow-up information was available until 31st of December 2016, and survival times were censored at 12 years post diagnosis. There were in total 303,792 individuals included in the analyses. We used 8 different models or approaches to estimate the LEL for this group of patients, as listed below, to illustrate various modelling choices. If not otherwise specified all estimations were based on flexible parametric models for excess mortality, with 5 degrees of freedom for the baseline cumulative hazard and 3 degrees of freedom for time-varying effects. The effect of age and calendar year were modelled continuously using splines with 4 degrees of freedom and interactions between age and year were included using splines with 2 degrees of freedom. All effects were allowed to be time-varying, i.e. we relaxed the proportional excess hazard assumption, which is important since the proportional hazards assumption is often violated in population based cancer studies. For the expected mortality and survival we used a population lifetable specific to England stratified by age, sex and calendar year. Extrapolations of excess mortality was based on the model parameters, and future expected mortality rates were assumed the same as in 2013 (the last year of available data in the population lifetable). Approach 1 The model and approach described above. Approach 2 The future expected mortality rates beyond 2013 were based on projected mortality rates in England. Approach 3 Cutoffs were used for age so that effect of age on excess mortality was the same for all ages up to 40, as well as all ages from 90 and above. Approach 4 A period approach was used, with period window 1st of January 2012 to 31st of Dec 2013, and calendar year was not included in the model. Approach 5 As 1, but 7 degrees of freedom for the baseline cumulative hazard and 4 degrees of freedom for time-varying effects. Approach 6 As 1, but 8 degrees of freedom for the baseline cumulative hazard and 5 degrees of freedom for time-varying effects. Approach 7 Deprivation status was included in the model, and a lifetable including deprivation was used. The same period approach as described above, and calendar year was not included in the model. Approach 8 As above, but a restriction was used for the effect of deprivation, so that the hazard ratios for deprivation were assumed constant after the last available follow-up.

## Results

### Expected mortality beyond the last year

**Fig. 3:**
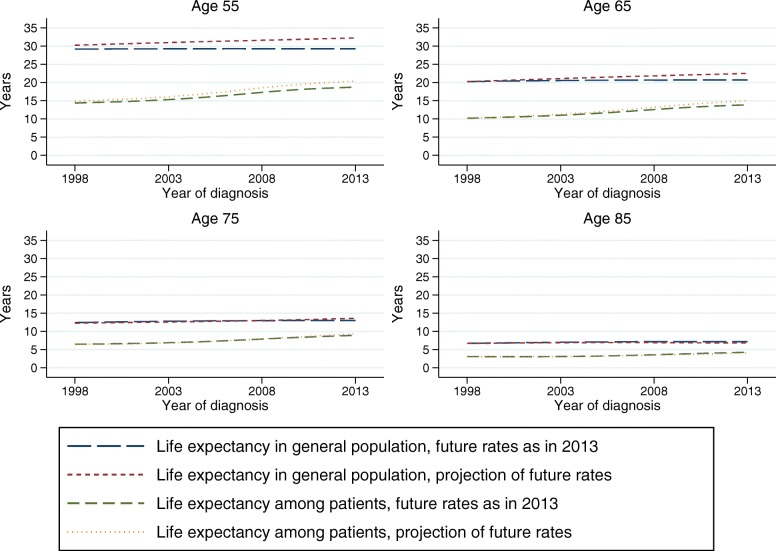
Estimated life expectancy for colon cancer patients, as well as general population, using population projections vs assuming constant mortality for expected survival beyond 2013

Different assumptions can be made regarding the future expected mortality rates, and mortality projections for a given population can often be obtained from official statistics bureaus. Alternatively, one can assume that the age- and sex-specific rates stays constant at the levels observed within the last available year, which is similar to how standard life expectancy is estimated. We used population mortality projections for England, produced by The Office for National Statistics, and compared the estimations of life expectancy using these projections to estimations based on assuming that the 2013 rates continued in the future. Figure 3 shows the life expectancy of female colon cancer patients across calendar year at diagnosis, for four selected ages, along with the life expectancy for females in the general population, from approach 1 and 2 as listed above. The two approaches for extrapolating expected mortality rates give slightly different values of life expectancy for later calendar years, and for younger patients, but the LEL difference is small. When we look at differences in LEL between population subgroups (e.g. deprivation or sex) the impact will be minimal.

Population mortality projections might not always be available, which might make it more practical to use the current rate within the latest year. The approach used might influence the younger ages more than older ages, and if possible we recommend to evaluate both approaches as a sensitivity analysis. Either way, it is important to always report which assumption has been made regarding the population mortality rate beyond the last year of observed data.

### Modelling age effects

**Fig. 4:**
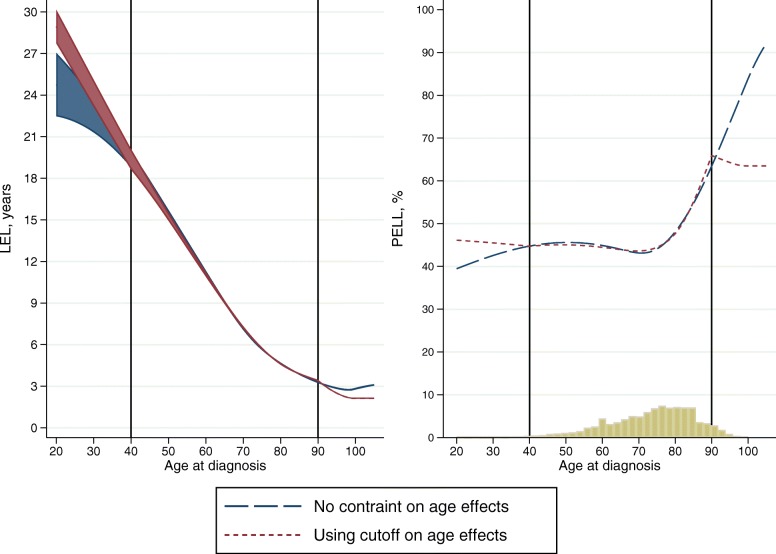
Loss in expectation of life and proportion of expected life lost, with different approaches for modelling the age effect

Age at diagnosis is commonly modelled by categorising age, and assuming that the excess mortality is constant within age groups. However, since mortality changes also within age groups, it is preferable to model age continuously e.g. by splines. Since the expected mortality rates, and life expectancy, changes continuously with age, it is even more important to model age continuously when the objective is to estimate the life expectancy or LEL. Due to few individuals diagnosed at young or old age, the results can be somewhat imprecise for these ages, and there can sometimes be problems with convergence of the models and the predictions can be unstable for the very young or very old. One solution for this is to assume that the effect of age on the excess mortality is the same for all ages below a certain cutoff, and similarly the same for all ages above a certain cutoff. Results based on approach 1 and 3 are shown in Fig. 4, where the LEL is presented across age for female patients diagnosed in year 2005. The cutoff values, age 40 and 90, are shown with vertical lines. The estimated LEL differ for younger and older ages, but the uncertainty is also higher in the younger ages, where the data is sparse. The PELL is also presented in Fig. 4, along with a histogram showing the age distribution among colon cancer patients in 2005. Even though the results differ in the youngest and oldest ages, the number of individuals within these ages is low, less than 6% of the patients are below 40 or above 90 at diagnosis. When summing up the total LEL among females diagnosed in 2005 the difference is negligible, 62404 for approach 1 and 62418 for approach 3, since the contribution from the few patients younger than 40 and older than 90 at diagnosis is small.

We recommend to use an approach such as approach 3 if needed for model stability, for example to avoid problems with convergence. This is even more useful when trying to fit models to many cancer sites to ensure that all models converge. Approach 3 can also be used as a sensitivity analyses, to see that the overall results are not sensitive to the model fit among the few young and old individuals. If the interest however, lies in the LEL for older individuals, this approach should not be used.

### Period approach vs modelling calendar year effect

**Fig. 5:**
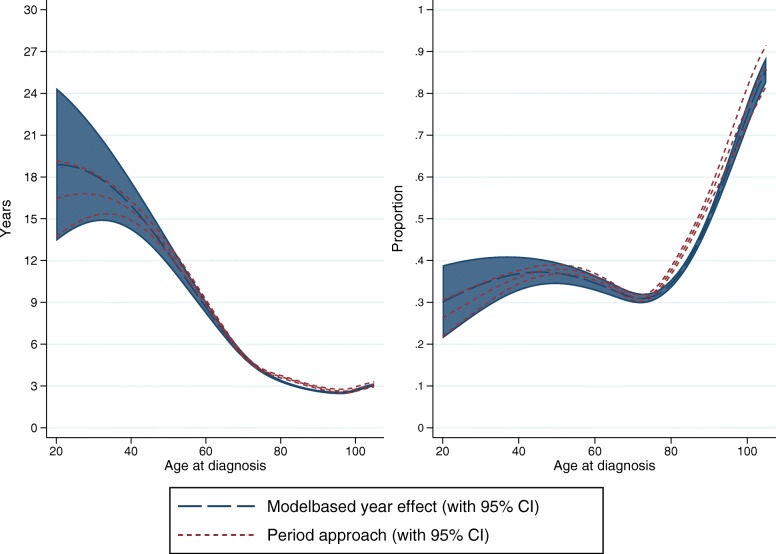
Period approach vs modelling calendar year effect

Period analysis can be used to obtain survival estimates for recently diagnosed patients, for which there is limited follow-up [17, 18]. Since improvement in cancer patient survival over calendar time is mainly observed in short-term survival, the yet unobserved long-term survival for recently diagnosed patients can be estimated from cancer patient survival diagnosed in earlier calendar periods, which is the approach used in period analysis. Alternatively, estimates can be obtained by extrapolating the calendar year effect from a model including year if diagnosis as a covariate. When temporal trends are of interest, the latter approach might be preferred, and the former approach might be preferred when the aim is estimation of LEL for recently diagnosed patients only. Results from the two approaches are compared in Fig. 5, where the LEL and PELL across age is plotted for female patients from approach 4 and for calendar year 2013 from approach 1. Even though there are some differences, especially for younger ages, the differences are generally small. If the interest is to estimate the total number of life years lost for a typical yearly colon cancer cohort, this can be estimated using the age distribution for a specific year. For 2013, the total number of life years lost for females is 58253 based on approach 1 vs 60097 based on approach 4, illustrating that the differences for the whole population is not great even if the difference can be large for the younger ages.

The choice between a period approach vs modelling calendar year often depends on if the interest is in primarily estimating the LEL for recently diagnosed patients, where the period approach would be used, or if the interest is in estimating temporal trends, where the calendar year modelling would perhaps be more appropriate. However, for the latter, it is important to model the effect of calendar year appropriately including important interaction effects.

### Knots, sensitivity analyses

**Fig. 6:**
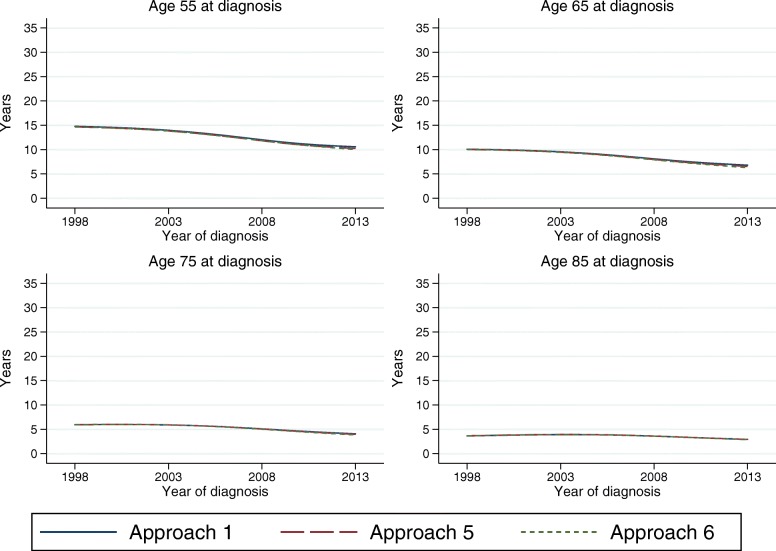
Sensitivity analyses of LEL estimates using different number of knots

When using splines it is important to perform sensitivity analyses, to evaluate the robustness to the number and placement of the knots. It has, however, been shown that the flexible parametric model is robust to the choice of knots [19]. Figure 6 shows the estimated LEL for female patients from model 1, 5 and 6, over calendar time for 4 selected ages. It clearly demonstrates that the LEL is robust to the knots. Even so, the different number of knots could lead to models that behave differently at the tail of the distribution of event times, and therefore extrapolate differently. This small differences are, as seen in Fig. 6, not so important when estimating the LEL at time of diagnosis, but can lead to larger differences in conditional LEL.

**Fig. 7:**
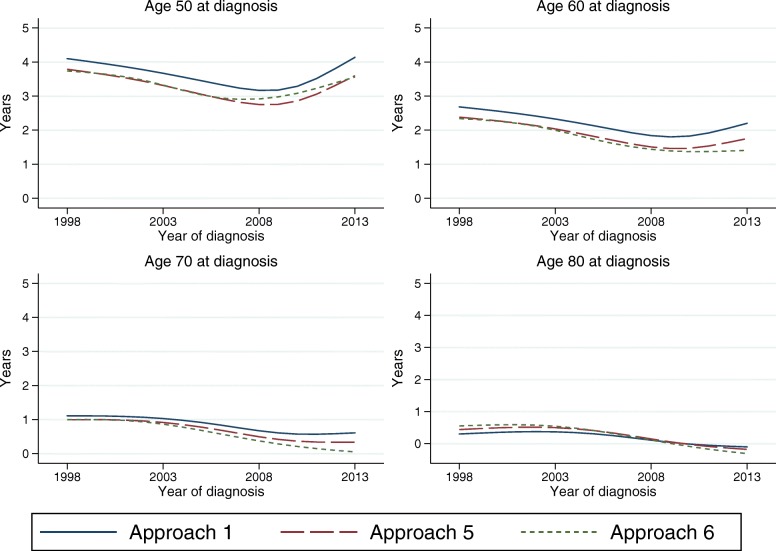
Sensitivity analyses of 5-year conditional LEL estimates using different number of knots

Figure 7 shows the 5-year conditional LEL from the three models, and the differences are slightly larger. However, the differences are still relatively small, e.g. the 5-year conditional LEL for age 50 at diagnosis in year 2008 ranges between 2.8 and 3.2 years. The 5-year conditional LEL is negative for older ages, indicating that older cancer patients that survive at least 5 years have a longer life expectancy than someone of the same age in the general population. This could be due to a healthy survivor effect, that those who survive are stronger and healthier and have more contact with the health care system than the general population. But it could also be due to the modelling assumptions, or that the population mortality rates are not appropriate in older ages.

Generally, the knots in a flexible parametric model are placed according to the distribution of event times, which, in studies of mortality among cancer patients, often means that there are few knots towards the end of follow-up time. It could therefore sometimes be useful to add an extra knot towards the end of follow-up, when extrapolating the survival. We recommend to always do sensitivity analyses to evaluate the effect of different number of knots and knot locations, even though this generally has a limited impact on the results, except possibly on conditional estimates.

### Extrapolating covariate effects

**Fig. 8:**
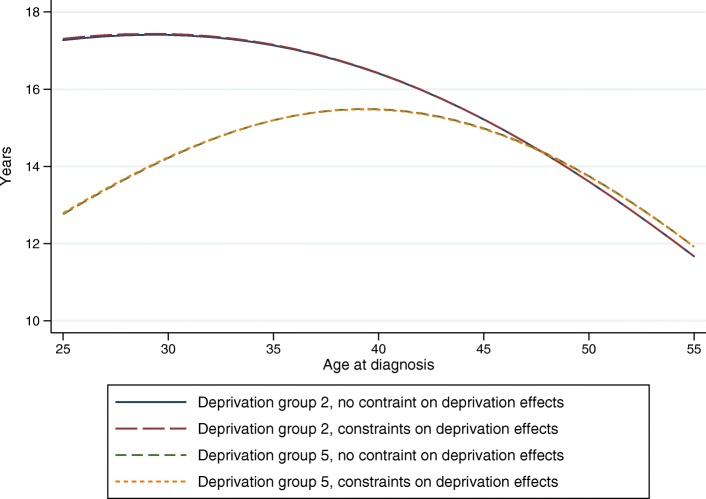
Extrapolating covariate effects

LEL can be a useful tool for quantifying differences in cancer patient survival by different groups, such as deprivation groups. Since the effect of deprivation on excess mortality often decreases by time, it is commonly modelled with non-proportional hazards. When extrapolating from a model with non-proportional hazards, as when estimating LEL, the time-varying effects are also extrapolated and can give rise to excess hazards functions that cross or increase/decrease at a non-believable rate after the end of follow-up. Crossing excess hazard functions can be prevented by constraining the extrapolated effects to be proportional beyond the available data but allowing for non-proportional hazards within the available follow-up. This was done within approach 8, and compared to a model without constraints (approach 7), and results of LEL for two deprivation groups are shown in Fig. 8 across age for female patients diagnosed in 2012. The results from the two approaches are very similar, partly since the the excess mortaliy is so low at this point, so different relative effects make little difference in absolute terms.

We recommend again that the approach of constraining covariate effects should be considered as sensitivity analyses, but it is unlikely to make too much difference as other cause mortality begin to dominate as time progress.

## Discussion

We believe that the LEL is a useful summary measure of cancer patient survival, and a good complement to other survival measures such as the often reported 5-year survival. LEL is easy to interpret, and it gives a measure of the disease burden and the impact of cancer on a patients life expectancy. In comparison, the 5-year survival is a measure of net survival, the proportion of patients that survive 5 years if the specific cancer would be the only possible cause of death. This is interesting when comparing groups or populations with different other cause mortality, but is not a good measure of disease burden. Estimation of LEL usually requires extrapolation of the survival function, since the cancer patients are not followed long enough for the survival to reach zero. An alternative is to estimate restricted mean survival, which does not require extrapolation, but is instead more difficult to interpret [20, 21]. Another measure similar to LEL is the years of life lost (YLL) [22–25]. The YLL is estimated for those that die in a set period, by comparing the age at death to the typical age at death for the population, with those that die at an older age than this reference value not contributing to the metric. The YLL measure does not rely on extrapolation, but has the limitation that the measure will contain a mix of patients diagnosed at different points in calendar time, so is not applicable to a certain cohort of cancer patients. The YLL measure may also rely on accurate cause of death information if those included in the analysis are defined by using only those that die due to cancer.

Estimation of LEL generally requires extrapolation of the survival function, and we have previsouly shown that the survival of the cancer patients can be satisfactorily extrapolated by making assumptions for extrapolation of the cancer mortality and the other cause mortality independently [7].However, when extrapolating survival from a model including covariates care has to be taken regarding the assumptions underlying the extrapolation. We have in this paper illustrated alternative ways for modelling covariate effects and different assumptions regarding the extrapolation. This is shown using data on on colon cancer in England. Generally, the different modelling assumptions and approaches give small differences in the estimates of LEL, however, the results can differ for younger ages and for conditional estimates.

## Conclusion

We recommend to always perform sensitivity analyses to evaluate the effect of the assumptions made when modelling and extrapolating survival to estimate the LEL.

## Data Availability

The data that support the findings of this study are available from Public Health England (https://www.gov.uk/government/publications/accessing-public-health-england-data/about-the-phe-odr-and-accessing-data), but restrictions apply to the availability of these data, which were used under license for the current study, and so are not publicly available.

## Abbreviations

LEL: Loss in expectation of life
PELL: Proportion of expected life lost
YLL: Years of life lost
